# Communication and coordination of care for people living with HIV: a qualitative study of the patient perspective

**DOI:** 10.1186/s12875-023-02243-x

**Published:** 2024-01-10

**Authors:** Sherridan Warner, Daniel Cheung, Ashleigh Condon, Juliet Cunningham, Jodie Bailie, Ariane Minc, Simone Herbert, Natalie Edmiston

**Affiliations:** 1 Faculty of Medicine and Health, The University of Sydney, Sydney, Australia; 2 University Centre for Rural Health, The University of Sydney, 61 Uralba Street, Lismore, NSW 2480 Australia; 3 School of Public Health, The University of Sydney, Sydney, Australia; 4 North Coast Sexual Health Service, Mid North Coast New South Wales Local Health District, Lismore, Australia; 5 School of Medicine, Western Sydney University, Campbelltown, Australia

**Keywords:** Multimorbidity, Primary care, Coordination of care, Patient experiences, HIV

## Abstract

**Background:**

There is growing consensus that primary health care (PHC) providers have an important role in providing holistic, preventative care for people living with human immunodeficiency virus (PLHIV). In regional Australia, HIV care is primarily delivered through specialist services, thus adequate coordination and communication between specialist and PHC professionals is crucial. This study aimed to explore patient experiences of the coordination of care and health care professional communication for PLHIV in regional Australia.

**Methods:**

Semi-structured interviews with PLHIV in a regional area of Australia were conducted in March to April 2022. Interviews were conducted via video conferencing, face-to-face, or via telephone call. Interviews were audio-recorded and manually transcribed. Transcripts were coded inductively and thematic analysis was conducted to explore perspectives on communication and coordination.

**Results:**

Thirteen participants were interviewed. Most participants were male, aged 50–70, were diagnosed with HIV more than ten years ago, and had been living in regional Australia long-term. Through qualitative analysis, themes emerged in the following areas: (1) Patient perception of care coordination; (2) Patient understanding of modality of communication; (3) Positive attitudes towards communication between healthcare professionals; and (4) Concerns for information sharing between healthcare professionals. Many participants highlighted lack of clarity around care coordination as a key issue in their healthcare, with some citing themselves as the primary care coordinator. Participants identified that coordination and communication between PHC professionals and specialist services are essential in the delivery of their health care, but some were hesitant for this to occur. Hesitancy was entrenched in some patients’ distrust of healthcare due to previous experiences of confidentiality breaches and stigma.

**Conclusion:**

This study identifies the need for clarity in coordination between health care professionals to deliver safe and effective HIV care, which may occur through care plans. Patient support for communication between healthcare providers may be strengthened by ensuring trust in the people and systems involved. Eliminating stigma in healthcare as well as building more trustworthy electronic-based communication technologies are essential components to trust-building between PLHIV and healthcare systems.

**Supplementary Information:**

The online version contains supplementary material available at 10.1186/s12875-023-02243-x.

## Introduction

Advances in the diagnosis and treatment of Human Immunodeficiency Virus (HIV) has led to a reduced HIV-associated mortality rate and more people living with HIV (PLHIV) [1]. PLHIV are an ageing population, with approximately 46% of PLHIV in Australia over 50 years old in 2019 [2]. With treatment, HIV can be characterised as a chronic illness with complex and unique multimorbidities, which may be a direct result of immunosuppression related illnesses, medication toxicity associated with antivirals, and generalised conditions associated with ageing [3]. In regional areas of Australia, the average age of PLHIV is older than in urban areas and multimorbidity is common [4].

PLHIV in Australia can access antiretroviral prescriptions from specialist physicians (including sexual health specialists, immunologists or infectious disease specialists), or primary health care (PHC) providers who have undergone training to prescribe antiretrovirals [5]. Access to these PHC providers in regional Australia is limited, meaning PLHIV in regional areas are more likely to need to seek specialist physician care [6]. Given the complexity of managing multimorbidity in PLHIV, care may best be delivered by specialist physicians in conjunction with PHC providers who may not be antiretroviral prescribers through a shared care model [7–9].

Shared care is defined as the coordination of specialists, PHC providers, and other health practitioners to deliver integrated care for a patient [5]. This model has been utilised in other health conditions, such as cancer survivorship and mental health [10]. Shared care allows patients to receive specialised treatment, while maintaining a good relationship with their PHC provider for multimorbidity management [11]. Shared care models in HIV have been evaluated by Australian state governments and other organisations, in which the need for better coordination between services was recommended [12, 13]. However, evidence is lacking in patient perspectives [14], particularly regarding attitudes to communication and care-coordination in a shared care model.

The success of shared care relies on coordination between PHC providers and specialists, and clear communication of each team member’s role and responsibility [15, 16]. Communication between healthcare providers may be in the form of letters, phone calls or sharing of results, and specific examples of the information required to be communicated may include changes to medications, new medical issues or hospitalisations. It is essential that this model of care is clearly understood by the patient and their caregivers [17]. Electronic health records are a means of bridging the gap in communication between specialists and PHC providers, by facilitating the integration of information, and allowing linkage to social support organisations [18–20].

Communication has been identified as a major facilitator in the success of shared care [21]. However, a clear strategy to support PHC and specialist communication for HIV care coordination is lacking. Consumer perspectives are required to ensure that any strategies implemented in the future align with patient values. This study aims to assess patient perspectives on communication and care coordination by PHC providers and specialists for PLHIV in a regional area.

## Methods

### Setting

In Australia, healthcare is accessible through Medicare, a universal health insurance scheme and through state-funded clinics. This study was conducted in a regional area of Australia, where in 2021, 532 people are accessing antiretrovirals, the majority via state-funded Sexual Health Services (SHS) [1, 6]. SHS provide specialist care, as well as access to nurse and social work services. Some PLHIV access antiretrovirals through a PHC provider trained to prescribe HIV medications although very few trained PHC providers are available in this region [6, 22]. A recent study of 329 PLHIV accessing a SHS demonstrated that approximately half of them engaged with a regular PHC, despite a high prevalence of multimorbidity [6].

### Study design

The study used an inductive qualitative approach which is deemed appropriate in situations where there is little prior research. Further, this approach is useful when exploring topics grounded in participants’ living experiences. We adopted a constructivist perspective, which assumes that neither data nor theories are discovered but rather are constructed based on the shared experiences of researchers and respondents [23]. Design and reporting of our study were guided by the Consolidated Criteria for Reporting Qualitative Research guidelines [24].

### Participants

Participants were recruited from the SHS, as well as through social media of a community HIV support organisation. Convenience sampling was used for recruitment, including a poster advertising the study, as well as verbal invitation by the health care provider at the end of telehealth consults if patients were unable to see the poster visually due to COVID-19 restrictions. Snowballing, whereby potentially interested participants were able to be identified from those already participating, was also used to recruit participants [25]. Due to the various recruitment and promotion strategies, we were unable to record the number of people aware of the opportunity to participate. Inclusion criteria were being willing and able to give informed consent, aged over 18 years and living or accessing HIV care in the region. Patients were compensated for their time with a $30 gift card, funded by Mid North Coast HIV and Related Programs Services. It was initially anticipated that fifteen participants would be required to satisfactorily reflect the experiences and views of consumers in this community. We recruited 11 male participants and began to see consistent themes in the data, however it was difficult to recruit from other groups such as women and those not actively engaging with healthcare and further attempts were considered unlikely to reach these groups.

### Data collection

This study was part of a larger study exploring patient perspectives of HIV care models in a regional area of Australia. Interviews were conducted by four medical students (AC, DC, JC, SW; three female, one male) undertaking a research project and collated for independent data analysis. There was no relationship between participants and interviewers prior to the commencement of the study, nor were interviewers involved in the clinics. Prior to the commencement of interviews, interviewers received training in interview skills and cultural safety, and attended discussions about general HIV care with specialists, and the lived experience of HIV and stigma with a person living with HIV.

Semi-structured interviews were conducted using a schedule developed in collaboration with sexual health specialists, with consultation of PLHIV (Supplementary File 1). Consistency between interview content was ensured by regular debriefing sessions between interviewers and in consultation with sexual health specialists. Interviews were conducted via a secure video-conferencing tool, face-to-face at the SHS, or via phone call. Only one interviewer and participant were present in each interview. Interviewers critically reflected on their own assumptions to promote a heightened awareness of listening to stories as openly as possible. Audio was recorded using a stand-alone recording device. No field notes were made during interviews. The average interview length was 52 min (range 36–88 min). No repeat interviews were conducted. Interviews were manually transcribed verbatim by the interviewer.

### Data analysis

Transcripts were loaded in NVivo qualitative data management software [26] for coding, searching and organising data. The lead author (SW) read all transcripts multiple times, making reflective notes in the process. Using an inductive approach, transcripts were openly coded by SW. Coding and subsequent thematic analysis were generated according to the research question; that is, it aimed to assess the patient perspective of communication and coordination of shared care for PLHIV in regional Australia. Examples of initial codes included “patient awareness of communication”, “coordination of care”, “modality of communication”, “lack of communication”, and “patient attitudes”. This was an iterative process, whereby codes were generated from one transcript, which then modified the coding of previous and future transcripts [27]. These initial codes were then used to postulate themes, defined as an idea or pattern that showed commonality between interviews and had significant meaning in relation to the research question [28, 29]. Themes were discussed as part of a member-checking process with the SHS team, comprised of sexual health specialists, counsellors and clinical nurse consultants, to ensure coherence of codes and thematic analysis. These themes were reviewed altogether to ensure that they supported the data, did not overlap, and that there were no themes missing. Themes were then defined and named.

### Ethics

This study was approved by the Northern New South Wales Human Rights Ethics Committee (approval #HREA324 2021/ETH11058) and received ACON^1^ research review approval prior to the commencement of this study.

## Results

Thirteen people were interviewed in this study; their attributes are summarised in Table 1. Almost all participants were male, and the majority of these participants received their HIV diagnosis over ten years ago, and have lived in a regional community for a large portion of their lives.

**Table 1:** Summary of interview participants.

Participants	13
**Gender**	
Male	11
Female	1
Non-binary	1
**Age (years)**	
50–59	6
60–69	7
**Indigenous**	
Yes	0
No	13
**Interview method**	
Face-to-face	5
Video Conference	4
Phone call	4
**Consistent regular PHC provider**	
Single consistent PHC provider	9
No consistent PHC provider	3
Multiple consistent PHC providers	1
**Time since diagnosis**	
< 2 years	0
2–10 years	1
> 10 years	12
**Time living in regional NSW**	
< 2 years	1
2–10 years	2
> 10 years	10
**Number of self-reported comorbidities**	
≤ 3	8
> 3	5
**Overall self-reported health rating**	
Poor	4
Fair	3
Good	6

Thematic analysis of interviews revealed themes in the areas of (1) patient perception of care coordination, (2) patient perception of communication, (3) positive attitudes towards communication, and (4) concerns for communication.

### Patient perception of care coordination

Most people were unsure who coordinated their care, with themselves or the specialist most involved. Most participants did not clearly identify a defined leader of their health care, and would not know who would coordinate a complex healthcare issue unrelated to HIV:

*“I think I’m kind of navigating it on my own, but that’s just within the um what I’ve experienced. Like I think if I got a complex problem that wasn’t just HIV related, involved other specialities, I mentioned [General practitioner, GP*^2^*] would probably. Oh well I don’t know actually whether or not [GP] or [specialist] would take the reins as far as coordinating stuff, I really don’t know”* Participant #10

Nine participants reported having a consistent regular PHC provider. Three participants had no consistent PHC provider, citing poor experiences such as issues with cost, trust, and large conglomerate medical practices as reasons for not engaging. One participant had two regular PHC providers, which they saw for different reasons. Further, most participants described a considerable amount of effort required to find a suitable PHC provider, and challenges in finding another when their PHC provider was no longer available. In the absence of a health practitioner as leader, most identified themselves as the key coordinator of care. Some participants described pro-actively adding other health care providers names to pathology request forms to facilitate communication. One participant pointed out that patient coordination may not be accurate, and may lead to inadequate care:

*“…If you haven’t got one or someone [overseeing care], then you’re relying upon the patient doing that. If the patient doesn’t know how to do that or just doesn’t have the opportunity, then there’s a risk of care failing”* Participant #12.

Where a health care practitioner was identified as a care coordinator or leader, this was often the specialist physician, as these are commonly the first and most regular healthcare provider that the patient will see:

*“Well, obviously [specialist] to date … when I first moved up here, I had to find a HIV clinic … [specialist] has kind of been directing traffic…”* Participant #11.

Another participant explained that their pre-diabetes was first detected by their specialist physician, during a six-monthly review including blood tests. One participant identified their GP as the major coordinator of their care, as they had several comorbidities that were also coordinated by the GP. Participants have experienced GP coordination of care for other chronic conditions such as diabetes, with three participants having completed a GP Management Plan^3^ as a means of team-based coordination of said illnesses. Where participants had this experience, they were more likely to have an understanding of the model of shared care for HIV and appreciate the importance of communication between providers.

### Patient perception of communication

Overall, most patients agreed that communication between healthcare providers was necessary for their care, but knowledge of this process varied. Some patients were explicitly aware of communication, as they had signed consent forms regarding information sharing when transferring into a new healthcare team. Other patients had not considered how their healthcare providers were communicating, and were not explicitly aware of it:

*“Well, I do think all of them should be talking together …as far as I know, there should be communication especially between the GP and all the other professionals in relation to my care.”* Participant #2.

Interestingly, some participants acknowledged that there was probably less communication occurring when HIV is well managed. While participants were aware on some level that communication between healthcare providers was occurring, they lacked a clear understanding of the specific modalities by which communication took place. When probed, participants listed a variety of communication methods, such as “electronic emails”, “occasional phone calls”, referral letters, and through pathology reports. Some participants recounted instances of their specialist informing them of communication with other healthcare providers:

*“But I can only go on what [specialist] says to me. [Specialist] just says ‘oh, I’ve had a report back from the haematologist’. So [specialist] is aware of [other specialist]’s results and comments.”* Participant #9.

Few participants volunteered perspectives on MyHealth Record (MHR), the Australian electronic health record^4^ [30]. Only one participant reported actively engaging with MHR, and appreciated the transparency afforded by MHR, allowing them to view communication. Two participants acknowledged that MHR made accessing medical records more convenient. However, other participants revealed that they had opted out of MHR due to privacy concerns:

*“… we do have a national one but the thing is, I feel, anyone can have access to it. And you don’t want to have anyone having access to your file, your medical history, or your medical presence… that’s not supposed to have it.”* Participant #7.

One participant expressed that they would be more confident in MHR if it was kept in physical form, such as on a portable hard drive. This highlights the importance of managing concerns around data sharing and privacy in PLHIV, as a marginalised population.

Participants identified areas where communication between their healthcare providers is insufficient for shared care. One participant proposed that there should be more structured communication, in the form of an annual multidisciplinary meeting involving all members of their healthcare team:

*“What if, once a year, my, I could have a telehealth conference with both of them … rather than relaying information from one place to another and then it coming back later when the doctors have had time to look at it…”* Participant #5.

Finally, the need for better communication structures between state healthcare systems was an important concern for people living in border areas, who split care between states.

### Positive attitudes towards communication between healthcare providers

Despite varying understanding of methods of communication between healthcare providers, participants expressed positive attitudes for communication as a means to achieving good healthcare. Communication between healthcare providers is valued highly for its additive value, safety and convenience. Patients often identified that communication and collaboration between healthcare providers results in better care overall when compared with care received by each team member alone, which can be defined as additive value. This was expressed as a benefit to shared care by many participants, where team communication resulted in better care:

*“It works well because when you’re talking to a doctor or something, you know you’ve got limited time, you may not get all the detail out or something important you’ve forgotten … little, correspondence and chats behind the scenes with all the other people it all comes together in one spot … you get a better, clearer picture. The team does. And I think I end up with better care.”* Participant #1.

Participants identified effective communication as important for safe health care, both physically and emotionally. One participant recounted an instance in which his healthcare providers communicated to conduct a medication review in order to prevent any adverse events:

*Interviewer: “So did you say you were taking four tablets a day?”*

*Participant: “Yeah, four tablets daily yes, and that’s been reviewed by…in Sydney by my GP, my doctor for HIV and my cardiologist and they discussed about the different drugs”* Participant #2.

A lack of communication between healthcare providers was identified as a potential risk to patient safety. When communication between healthcare providers relies on a patient’s recount of information, that information may not be accurate, which has implications in clinical decision making and patient safety.

Communication and collaboration in HIV care was also identified to influence emotional wellbeing. Participants suggested that it allows them to feel connected and cared for by a team, particularly when information is shared tridirectionally; that is, between two healthcare providers and the patient:

*“Well the security I guess… knowing what’s happening with you and whether you’re doing things right in your life, cause lifestyle has a huge impact on your health and it’s just important to keep things under control. ‘Cause the communication’s there…I always feel like I’m informed and secure with that information”* Participant #8.

Convenience of communication was a commonly identified benefit amongst participants. Communication between healthcare providers alleviates the burden of information sharing and coordination of care from the individual:

*“I have a long-term relationship with medical people, it’s really good because you don’t have to explain everything every time and they understand, they’ve got the background there… and it’s not as confronting either, with me anyway.”* Participant #1.

When communication is lacking, patients may feel as though they are having to relay information more often, which can be frustrating. Failed communication between healthcare providers may result in over testing for the patient:

*“I just have to take a deep breath sometimes and put up with being asked the same twenty questions by five doctors”* Participant #4.

### Concerns about communication between healthcare providers

Communication concerns were minimised when trust was established but patients’ previous experiences perpetuated hesitancy. Some participants did not take issue with information sharing; they trusted their healthcare providers to share information with care and respect, just as they are cared and respected in their healthcare interactions:

*“No, no, I’m good, I think it’s a great thing, I believe that I have trust in the ethics and privacy that my health care team provides. I feel like they’re all very reputable and they look after my information as much as they look after me.”* Participant #5.

However, some participants recounted traumatic experiences of stigma in healthcare. For some, this led to distrust, either in an individual healthcare provider, or the healthcare system and consequently, hesitancy in the sharing of their medical information within the healthcare system:

*“And [specialist] is fine, I trust [specialist] 100%. The other’s I don’t. That sharing is going to my GP who I don’t trust”* Participant #6.

Privacy breaches were also a concern for some participants regarding information sharing within the healthcare practice, specifically inadvertent breaches of confidentiality by administrative staff. Hesitancy was also linked to a lack of autonomy in information sharing; participants expressed concern where their HIV status was disclosed to other healthcare providers without their choice:

*“I mean the thing about shared care is it’s not necessarily involving me in the choice of those people is it?…It’s one doctor deciding to involve other doctors without my say”* Participant #4.

## Discussion

This study highlighted a lack of clarity in the roles of each healthcare provider in the patient’s HIV care, with participants often identifying themselves as the main coordinator of their care. The responsibility of navigating information sharing and the coordination of healthcare services can be burdensome for patients engaged in shared care [31]. In study of 1400 PLHIV in London, many PLHIV regarded their specialist as the main care coordinator, and felt more comfortable with their specialist managing comorbidities compared with PHC providers [21]. This is in keeping with the findings of this study, in that specialist physicians tended to drive coordination of care due to being the first and most frequent healthcare provider that PLHIV see. This facilitates an ongoing relationship of trust and mutual respect between patient and specialist, which may allow the patient to feel most comfortable with their specialist coordinating their care, compared to a PHC provider who they do not see often.

Participants identified that communication between healthcare professionals allowed for safer and more effective HIV care. This has been shown in an interdisciplinary care model in which pharmacist communication with healthcare providers in HIV care led to better multimorbidity management, and more cost-effective care [32]. Patients value interdisciplinary care when they have confidence in communication between their healthcare providers, and structural barriers to communication have been identified as a major reason for interdisciplinary care failing [21]. Structural barriers to communication identified in this study included those preventing communication across states. The lack of consistency and accessibility of health information exchanges across different jurisdictions in Australia significantly impacts the ability of healthcare providers to coordinate care, and has been identified as a priority of the National E-Health Transition Authority [33].

Trust in the healthcare provider and the healthcare system was a major determinant of the patient’s perspective of communication between healthcare providers. Trust is intrinsically linked to a patient’s previous experiences of stigma in healthcare [34]. Stigma in healthcare settings may manifest as confidentiality breaches, humiliation and even refusal to treat [35, 36]. Past experience of stigma has been shown to impact engagement in health services, as individuals may anticipate stigma, and express distrust in healthcare provider and systems [37]. This was reflected in our study, whereby those who had previous traumatic experiences expressed hesitancy in communication between healthcare professionals, due to a lack of trust. Interestingly, those who felt that they were actively involved in the communication and coordination of their care seemed to be more trusting of this information sharing. Patient involvement in communication is highly valued in other shared care models, such as cancer survivorship [31]. Tridirectional sharing – between two healthcare providers and the patient – of information facilitates trust and allows the patient to feel some control over the sharing of potentially stigmatising information such as HIV status [38]. Health care providers and agencies including E-Health agencies have a responsible to demonstrate trustworthy behaviour, such as providing patient control, to build trust and enable safe care co-ordination.

Regarding trust in electronic health records for information sharing, there was hesitancy to engage due to distrust in electronic systems due to the risk of confidentiality breaches. In Australia, the national rollout of MHR was received with mixed opinions by PLHIV and supporting organisations, who have encouraged “opting out” due to privacy and criminalisation concerns [39]. While amendments have been made to protect vulnerable populations from privacy breaches within MHR, there are still significant concerns for many consumers, and little is known about how this has directly affected PLHIV [40]. However, recent literature has demonstrated that knowledge about MHR remains low in vulnerable populations such as PLHIV [41, 42]. In this study, those who expressed hesitancy also acknowledged utility in these systems. Previous studies showed gradual confidence in the security of technologies, as well as acknowledgement of their benefit in the context of their care; that is, the risks of confidentiality breaches were outweighed by the utility of electronic health records [43]. Demonstrated utility of and education regarding electronic health records such as MHR in Australia in coordinating care, may facilitate patient acceptance.

This study has provided unique perspectives from PLHIV in regional Australia. The literature has historically underserviced this population, with many consumer-focussed studies being conducted in metropolitan areas [44]. Regional communities have unique healthcare needs due to differences in access to care, as well as societal and cultural differences [45]. Therefore, our study is an important contribution to the field of literature, giving voice to a unique and underrepresented population.

Limitations of this study include lack of generalisability. There is a documented disparity in healthcare experiences between different genders, as well as between Indigenous and non-Indigenous Australians, and therefore future studies should endeavour to sample more diverse participants [46, 47]. Further, this study was unable to access the perspectives of those who are not currently engaged in HIV care or unwilling to disclose their HIV status. This population may have unique concerns about communication and information sharing between healthcare providers, and future research should aim to engage these PLHIV to broaden the perspectives obtained.

Multimorbidity is significant and increasing issue for PLHIV and health care systems need to evolve to meet this need [7]. When shared care is utilised, having clearly defined roles in the healthcare team, as well as more formalised communication channels, from the commencement of a patient’s HIV care would facilitate open communication when needed. Care coordination agreements have proved to be successful in HIV care where there was established communication channels and strong working relationships [48]. Existing GP Management Plans for PLHIV could be adapted to include more information about the role of team members, and how/when communication should occur [5, 49]. While participants often identified their specialist to be their main coordinator, literature suggests that the specialist may not be best placed to coordinate overall care [50]. Engaging a formal care coordinator may be a potential solution to care coordination for PLHIV. In a shared care model for chronic haematological malignancies, a clinical nurse specialist was appointed as care coordinator, which facilitated timely communication between healthcare providers, and collaboration within the healthcare team, as well as coordination with the patient to provide clarification around care management [11]. As well as nurses, peer support has important roles to play in enabling care co-ordination, particularly through the ability to address the persistent impact of stigma on engagement with health behaviours [51, 52]. Further research is needed from the perspectives of healthcare providers to ascertain who is best suited to lead HIV care coordination.

Shared care is received positively by PLHIV when there is excellent communication between PHC providers and specialists; patients value their PHC providers deferring to specialist physicians for consultation in making non-HIV clinical decisions, likely due to the established trusting relationship between themselves and their specialist [21]. However, issues included lack of confidence in the quality of communication between healthcare providers – for example receiving contradictory advice, or acting as a “messenger” – and structural barriers that prevented communication [21].

## Conclusion

The study found PLHIV in a regional area valued communication between health care providers for its additive value, safety and convenience. There is a lack of understanding around the communication and coordination of healthcare and concerns about stigma and confidentiality, however specialists’ support for shared care for patients with multimorbidity alleviated concerns.

### Electronic supplementary material

Below is the link to the electronic supplementary material.

Supplementary Material 1

## Data Availability

The complete datasets supporting the conclusions of this article are not available openly due to the sensitive nature of the data and the consent provided for participation in the specific study. Access to de-identified data may be discussed with the corresponding author Dr Natalie Edmiston, as limited data may be made available for similar projects subject to ethic review, as extended consent was obtained.

## Footnotes

1 ACON is a New South Wales community organisation that aims to support people living with HIV and the LGBTQ + community. More information on ACON can be found at https://www.acon.org.au/.

2 GPs (General Practitioners) are primary healthcare providers in Australia. Often, they are the first point of contact with healthcare for a patient, and generally coordinated healthcare needs of a patient.

3 A GP Management Plan is a unique feature of the Australian healthcare system, and involves a written set of information about what is needed to manage chronic or complex conditions. It is prepared by the patient’s regular primary healthcare provider and is akin to ‘a plan of action’ to help coordinate care, identifying health and care needs and sets out the services to be provided by the general practitioner and the actions individuals can take to help manage conditions. More information on GP Management Plans can be found at https://www1.health.gov.au/internet/main/publishing.nsf/Content/mbsprimarycare-chronicdisease-pdf-infosheet.

4 My Health Record (MHR) is an Electronic Health Record that is designed to allow health information sharing between healthcare professionals and the patient. MHR operates on an “opt-out” basis, whereby patients create a password-protected account that they can delete at any time. Information available on MHR includes allergies and drug reactions, hospital discharge summaries, radiological and pathology investigations, prescription and dispense information, specialist letters, and medicolegal documents such as advanced health directives. Patients can access their own MHR via website or mobile app. Healthcare providers can access patient MHRs through the National Provider Portal. More information on MHR is available at https://www.digitalhealth.gov.au/initiatives-and-programs/my-health-record?gclid=Cj0KCQjwocShBhCOARIsAFVYq0gw_yslyCuhbTnas0wgLLrNtqaF-2mSi3q7mBMr58lTPVzfipOrbu0aAm_WEALw_wcB&gclsrc=aw.ds.

## List of abbreviations

GP: General Practitioner
HIV: Human Immunodeficiency Virus
MHR: My Health Record
NSW: New South Wales
PHC: Primary Health Care
PLHIV: People Living with HIV
SHS: Sexual Health Service
