# Supplementary material for: Communication and coordination of care for people living with HIV: a qualitative study of the patient perspective
Source: BMC Prim Care. 2024 Jan 10;25:19. doi: 10.1186/s12875-023-02243-x (PMC10777490; doi:10.1186/s12875-023-02243-x)
Supplement: Supplementary file 1 — Supplementary Material 1 [file 12875_2023_2243_MOESM1_ESM.docx]

## Supplementary File 1: Interview guide

### Section 1

Would you like to tell me a little about how long you have been living with HIV and where you have been living during this time?

How long have you been living in xx?

Besides HIV, what other health conditions are you dealing with? *Probes – heart disease, mental health, liver disease, memory problems?*

What different health care providers do you currently see? Probes – *HIV/Sexual Health doctor, HIV/Sexual Health counsellor, nurse, GP, other counsellor, physio, chiro, dentists*

How would you rate your current health? *Very poor, poor, fair, good, very good.*

What other supports do you have to help you live with HIV?

GP care access

What has been your experience of accessing GPs? *Probes – regular GP vs non regular, number of different GPs*

If yes, what do you like about having GP care? What problems do you experience in using a GP? *Probes* – *benefits, transport, time, cost, information/communication*

If no, would you like to have a GP? What would be good things about having a GP? What has prevented you from using a GP? *Probes – perceiving need, suitability of GPs, transport, time, cost, information/communication*

Other service access

Are there any difficulties for accessing any other services? *Probes – distance, cost, appointment availability, stigma, disclosure*

### Section 2

Have you heard the term shared care? What does it mean to you?

By shared care we mean that health practitioners are working together to provide your health care in a co-ordinated manner, usually involving a GP, a specialist and other providers.

Do you have shared care for your HIV?

If yes, can you describe what that looks like for you?

In the last few years, xx HIV services have moved to encourage shared care where possible. How has this affected/impacted you?

To what degree do you feel like you have a choice in how you receive care?

What do you think sexual health services can do to improve shared care with GP in the future?

### Section 3

What health care do each of the different health practitioners provide for you?

How do you decide what health care issues each practitioners helps you with?

How do the various health care providers communicate with each other?

Do you have concerns about the communication between health care providers? *Probes – too much communication, not enough, consent for sharing of information?*

What is your role in your care?

If you see a number of health services, who helps co-ordinate or navigate that process?

### Section 4

What are the most valuable aspects of the care that you receive from your care providers?

Are there aspects of care that you receive, or have received, that you think is of little or low value? *Probes – clinical visits, blood tests*

If yes, why do you think low value care is happening? Does having more than one person involved in your care contribute to receiving low value care?

How does your care contribute to your wellbeing?
